# Supplementary material for: Anti-Zeno purification of spin baths by quantum probe measurements
Source: Nat Commun. 2022 Dec 6;13:7527. doi: 10.1038/s41467-022-35045-3 (PMC9726817; doi:10.1038/s41467-022-35045-3)
Supplement: Supplementary file 1 — Supplementary_Information [file 41467_2022_35045_MOESM1_ESM.pdf]

Supplementary Information: Anti-Zeno purification of spin baths by quantum probe measurements

Durga Bhaktavatsala Rao Dasari,[1] Sen Yang,[2] Arnab Chakrabarti,[3]
Amit Finkler,[3] Gershon Kurizki,[3] and Jörg Wrachtrup[1]

[1] *3. Physics Institute, University of Stuttgart,*
Center for Applied Quantum Technologies, IQST,
MPI for Solid State Research, Stuttgart 70569, Germany.
[2] *Department of Physics, The Hong Kong University of Science and Technology,*
Clear Water Bay, Hong Kong, China.
[3] *AMOS and Department of Chemical and Biological Physics,*
Weizmann Institute of Science, Rehovot, Israel

Supplementary Note 1. DYNAMICS OF PROBE ELECTRON SPIN COUPLED TO NUCLEAR SPIN BATH

A. Probe-bath interaction Hamiltonian in the star configuration for non-interacting nuclear spins

In the absence of an external magnetic field, upon neglecting the zero-field splitting, the interaction Hamiltonian for the probe spin with Pauli operator $\vec{S}$ resonantly coupled to a collection of non-interacting nuclear spins with angular momentum operators $\{\vec{I}_k\}$ is given in the laboratory frame, by

$$H = \sum_k \gamma_k \,\widetilde{g}_k \left[3\,(\mathbf{S}\,.\,\mathbf{r_k})(\mathbf{I_k}\,.\,\mathbf{r_k}) - \mathbf{S}\,.\,\mathbf{I_k}\right]. \tag{1}$$

Here $\mathbf{S}$ is located at the origin, $\mathbf{r_k}$ is the kth nuclear position, the dipole-dipole couplings are $\gamma_k\,\widetilde{g}_k$, where $\widetilde{g}_k = \gamma_e/r_k^3$, γ_e , γ_k denote the magnetic moments of the electron and the kth nuclear spin respectively [1].

Since the degeneracy in the NV electron spin-space is not broken by any external field, we can choose the z-axis of our laboratory frame to be parallel to the quantization axis of S. With this choice, the Hamiltonian in Supplementary Eq. (1) becomes

$$H = \hat{S}^z \sum_k \gamma_k \,\widetilde{g}_k \left[3\,r_k^z\,(\mathbf{I_k}\,.\,\mathbf{r_k}) - I_k^z\right], \tag{2}$$

where $\hat{S}^z$, I_k^z and r_k^z denote z components of the vectors $\mathbf{S}$, $\mathbf{I_k}$ and $\mathbf{r_k}$ respectively. Defining a new vector $\mathbf{g_k}(r)$ by the components of the hyperfine coupling in Supplementary Eq. (2), we arrive at the Hamiltonian in Eq. 1 of the Main Text. A factor of $\frac{1}{2}$ obtained from the representation of $\hat{S}^z$ in the $|e(g)\rangle$ basis is absorbed in $\mathbf{g_k}(r)$.

B. Conditional evolution of a non-interacting spin bath

The time evolution operator under the interaction Hamiltonian H in Eq. 1 of the Main Text is simply $U_{S+B}(t) = \exp[-i\,H\,t]$. For the non-interacting spin bath and in the absence of an external magnetic field, the basis $|\pm\rangle = \frac{1}{\sqrt{2}}\left[\,|e\rangle \pm |g\rangle\,\right]$ used in the Main Text is degenerate, so that the probe undergoes pure dephasing under the influence of the bath noise, i.e. random mixing of the $|+\rangle$ and $|-\rangle$ states. The time evolution operator, in this case, can be written as

$$U_{S+B}(t) = \cos(\hat{B}t)\Big[|+\rangle\langle+| \;+\; |-\rangle\langle-|\Big] - \mathrm{i}\,\sin(\hat{B}t)\Big[|+\rangle\langle-| \;+\; |-\rangle\langle+|\Big] \tag{3}$$

The initial probe density matrix is $\rho_S(0) = |+\rangle\langle+|$, while the initial bath density matrix $\rho_B(0)$ is given by Eq. 4 of the Main Text. The density matrix of the probe + bath, $\rho(t)$, at

time t can be written as

$$\rho(t) = U_{S+B}(t)\ \rho_S(0) \otimes \rho_B(0)\ U^{\dagger}_{S+B}(t)$$

$$= |+\rangle\langle+| \otimes \cos(\hat{B}t)\ \rho_B(0)\ \cos(\hat{B}t) +\ \mathrm{i}\ |+\rangle\langle-| \otimes \cos(\hat{B}t)\ \rho_B(0)\ \sin(\hat{B}t)$$

$$-\ \mathrm{i}\ |-\rangle\langle+| \otimes \sin(\hat{B}t)\ \rho_B(0)\ \cos(\hat{B}t) + |-\rangle\langle-| \otimes \sin(\hat{B}t)\ \rho_B(0)\ \sin(\hat{B}t). \tag{4}$$

S projection onto the $|+\rangle$ state yields the conditional bath evolution

$$\begin{aligned}
\rho^{+}_{B}(t) &= \cos(\hat{B}t)\ \rho_B(0)\ \cos(\hat{B}t) \\
&= \frac{1}{2}\Big[e^{i\,\hat{B}\,t} + e^{-i\,\hat{B}\,t}\Big]\ \rho_B(0)\ \frac{1}{2}\Big[e^{i\,\hat{B}\,t} + e^{-i\,\hat{B}\,t}\Big] \\
&= \frac{1}{2}\Big[\hat{U}_e(t) + \hat{U}_g(t)\Big]\ \rho_B(0)\ \frac{1}{2}\Big[\hat{U}_e(t) + \hat{U}_g(t)\Big]^{\dagger} \\
&= V_{+}(t)\ \rho_B(0)\ V^{\dagger}_{+}(t).
\end{aligned} \tag{5}$$

Similarly, S projection onto the $|-\rangle$ state yields the conditional bath evolution

$$\begin{aligned}
\rho^{-}_{B}(t) &= \sin(\hat{B}t)\ \rho_B(0)\ \sin(\hat{B}t) \\
&= -\frac{1}{2}\Big[e^{i\,\hat{B}\,t} - e^{-i\,\hat{B}\,t}\Big]\ \rho_B(0)\ \frac{1}{2}\Big[e^{i\,\hat{B}\,t} - e^{-i\,\hat{B}\,t}\Big] \\
&= \frac{1}{2}\Big[e^{i\,\hat{B}\,t} - e^{-i\,\hat{B}\,t}\Big]\ \rho_B(0)\ \frac{1}{2}\Big[e^{-i\,\hat{B}\,t} - e^{i\,\hat{B}\,t}\Big] \\
&= \frac{1}{2}\Big[\hat{U}_e(t) - \hat{U}_g(t)\Big]\ \rho_B(0)\ \frac{1}{2}\Big[\hat{U}_e(t) - \hat{U}_g(t)\Big]^{\dagger} \\
&= V_{-}(t)\ \rho_B(0)\ V^{\dagger}_{-}(t).
\end{aligned} \tag{6}$$

C. Probe-bath interaction Hamiltonian for an interacting-spin bath

For a probe spin resonantly coupled to one of the spins (labeled as 1) of a dipole-dipole interacting-spin bath, the full Hamiltonian has the following form, upon taking the energy origin to be at the resonant frequency

$$H = g_1 \sum_i \mathbf{S_1}.\mathbf{I_i} + \Big[\sum_{i,j} J_{i,j} I^{+}_{i} I^{-}_{j} + h.c\Big], \tag{7}$$

where $\mathbf{S_1}$ is the Pauli operator pertaining to spin 1, g_1 is the corresponding probe-spin dipolar coupling and the second term describes the interaction of spins i, j, via the dipolar coupling $J_{i,j}$.

In principle, one can always diagonalize the second term and transform the Hamiltonian to the simplified form [2]

$$\widetilde{H} = \widetilde{J}\Big[c^{\dagger}_{S} \sum_k \phi_k\, b_k + h.c\Big], \tag{8}$$

where $\widetilde{J}$ is the effective coupling, $c_S^\dagger$ is the fermionic probe-excitation (raising) operator, $\{b_k\}$ are the bath-eigenmode deexcitation (lowering) operators and ϕ_k are the corresponding expansion coefficients. For the nearest-neighbour spin-spin interaction in a one-dimensional spin-chain configuration known as the magnon model, $\phi_k = \sqrt{\frac{2}{N+1}\sin\left[\frac{k\pi}{N+1}\right]}$ (cf. ref. 3 of the Main Text). For more intricate spin-bath geometries there are no general expressions for ϕ_k and the corresponding spin-bath eigenvalues ϵ_k, but they are obtainable numerically.

D. Conditional evolution of an interacting-spin bath

The degeneracy of the symmetric and antisymmetric superposition states of the probe $|\pm\rangle = \frac{1}{\sqrt{2}}[\,|e\rangle + |g\rangle\,]$ is broken by coupling to an interacting-spin bath, so that the probe no longer undergoes pure dephasing due to couplings to such a bath. We may now measure the probe in the $|e(g)\rangle$ basis, thereby projecting the S+B state onto the corresponding bath state at time $t = \tau$. Thus, for $|e\rangle$ or $|g\rangle$ projections, $\rho_B^{e(g)}(t) = \langle e(g)|e^{-i\widetilde{H}\tau}\,\rho_B(0)\,e^{i\widetilde{H}\tau}|e(g)\rangle$. We may also project the probe on the $|\pm\rangle$ basis, even though it is now non-degenerate.

The steering of the bath evolution via a sequence of $|+\rangle$ and $|-\rangle$ projections or $|e\rangle$ and $|g\rangle$ projections defines CTs analogous to those discussed in the Main Text. Yet the resulting bath dynamics and its steady states may be drastically different (see Sec. IV).

Supplementary Note 2. SELECTIVE (SINGLE-SHOT) PROBE MEASUREMENT EFFECTS ON A NON-INTERACTING BATH VIA THE KK FORMULA

If we perform a selective measurement on the probe spin e.g. project it onto the basis state $|+\rangle$, the bath state collapses to (see Main Text)

$$\rho_B^{(1+)}(\tau) = \frac{1}{\mathcal{N}_{1+}}\sum_j P_j(0)\ \cos^2(\omega_j\tau)\ |j\rangle\langle j| \quad ; \quad \mathcal{N}_{1+} = \sum_k P_k(0)\ \cos^2(\omega_k t). \tag{9}$$

Similarly, for a projection onto the $|-\rangle$ state, the bath state collapses to

$$\rho_B^{(1-)}(\tau) = \frac{1}{\mathcal{N}_{1-}}\sum_j P_j(0)\ \sin^2(\omega_j\tau)\ |j\rangle\langle j| \quad ; \quad \mathcal{N}_{1-} = \sum_k P_k(0)\ \sin^2(\omega_k\tau). \tag{10}$$

A conditional trajectory (CT) in parameter space is here defined by a successful sequence of m projections of the probe, n out of which are on the $|-\rangle$ state and $m-n$ on the $|+\rangle$ state. As long as we keep τ fixed, the order of projections on $|+\rangle$ or $|-\rangle$ is immaterial, i.e. they can be permuted at will. For such a CT the final density matrix of the bath is given by,

$$\rho_B^{\{n,(m-n)\}}(\tau) = \frac{1}{\mathcal{N}_{n,(m-n)}(\tau)}\sum_j P_j(0)\ \cos^{2(m-n)}(\omega_j\tau)\ \sin^{2n}(\omega_j\tau)\ |j,\rangle\langle j| \tag{11}$$

$$\mathcal{N}_{n,(m-n)}(\tau) = \sum_k P_k(0)\ \cos^{2(m-n)}(\omega_k\tau)\ \sin^{2n}(\omega_k\tau), \tag{12}$$

$\mathcal{N}_{n,(m-n)}(\tau)$ being the overall normalization factor for this CT, which is denoted by $M_{n,m}$ in Eq. 8 of the Main Text.

The probability distribution for the bath state modified by this CT is

$$\tilde{P}_j(\tau) = \frac{P_j(0)}{\mathcal{N}_{n,(m-n)}(\tau)} \cos^{2(m-n)}(\omega_j\tau) \sin^{2n}(\omega_j\tau). \tag{13}$$

The distribution of $\tilde{P}_j(\tau)$ is approximately the bath spectrum $P(\omega_j)$ in Fig. 1(c). The cosine-sine rational polynomial in Supplementary Eq. (13) plays the role of the filter function $F(\tau, \omega)$ in the KK formula (Eq. 10 in the Main Text).

Supplementary Note 3. PURIFIED BATH SPECTRUM

A. Numerical results

We consider an N-spin-1/2 bath and m single-shot measurements. In order to use dimensionless parameters for simulation, we scale the bath-state eigenvalues by the rms coupling $\bar{g} = \sqrt{\sum_k g_k^2}$. The bath eigenfrequencies are to be determined from linear combinations of the couplings g_k that are typically random. Supplementary Fig.1 and Supplementary Fig.2 show the bath spectra for $N = 4$ and $N = 10$ spins after $m = 28$ and $m = 100$ measurements, respectively. The top left panels are for $n = 0$ (i.e. all $|+\rangle$ detection), for different values of $\bar{g}\tau$. In Supplementary Fig.1 we have assumed that $\left(\frac{\omega_j}{\bar{g}}\right)$ vary linearly within a period of $-\pi \leq \frac{\omega_j}{\bar{g}} \leq \pi$ in steps of π/n_b, where $n_b = 2^N$ is the bath Hilbert-space dimension. In the top left panel of Supplementary Fig.1 we see that for $\bar{g}\tau \geq 0.1$, the bath-spectrum becomes increasingly pure with the growth of $\bar{g}\tau$. The maximum peak amplitude is obtained for $\bar{g}\tau = 0.8$. This signifies an Anti-Zeno (AZE) cooling of the bath spectrum. Supplementary Fig.1 is well in agreement with the experimentally obtained post-measurement ODMR spectrum shown in 2(c) of the Main Text. In Supplementary Fig.1 and Supplementary Fig.2 (top right panels) we find that in the AZE limit, as n increases, the two major peaks in the purified bath spectrum shift from the center, toward the spectral edges. For some intermediate values, we have more than two peaks with reduced overall purity.

We repeat this analysis in the Zeno regime with $\bar{g}\tau = 0.05$ and show the resulting spectra in Supplementary Fig.1 (bottom panel). Notice that in this case the orange curve almost overlaps the black curve, as confirmed by Supplementary Eq. (16) below. For $n \neq 0$, the spectrum again becomes highly pure with increasing n. However, in this case, the peaks appear near the spectral edge. Similar spectra for an $N = 10$ spin-1/2 bath is shown in Supplementary Fig.2.

B. Bath states via probing in the Anti-Zeno and Zeno regimes: analytical results

Rewriting Supplementary Eq. (11) as

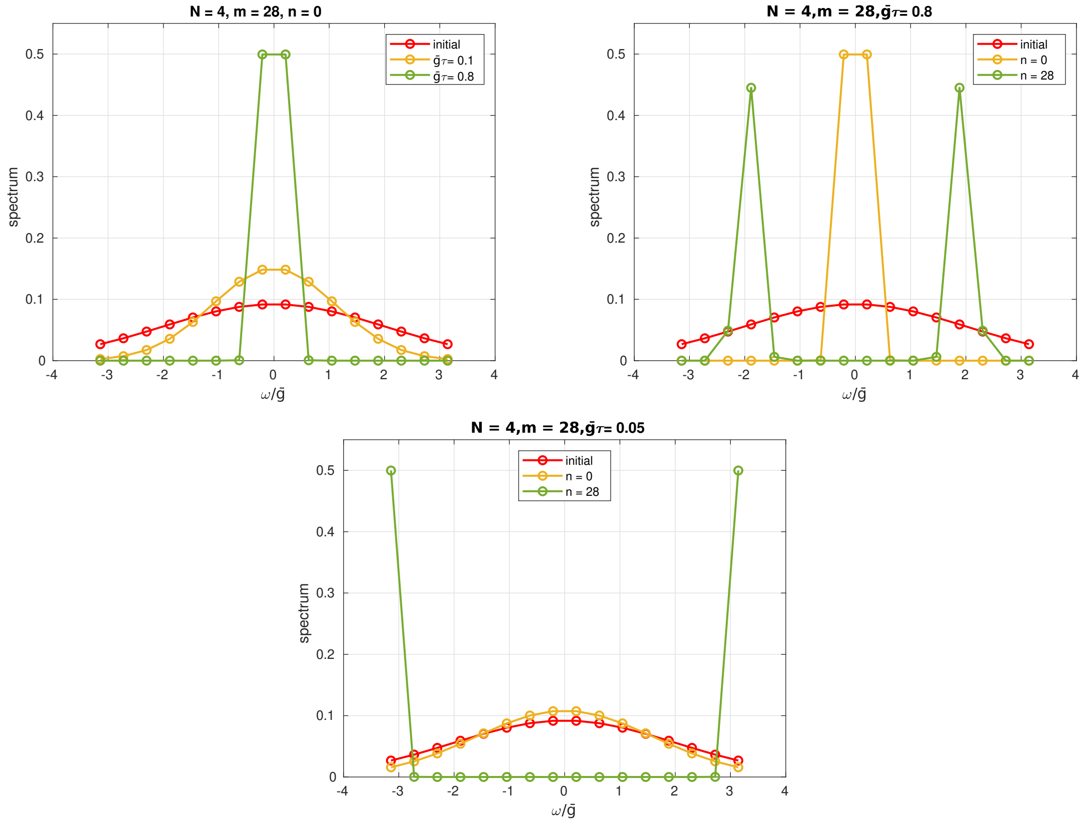

Figure Supplementary Fig.1. Spectrum, $P(\omega)$ of a 4-spin-1/2 bath, for $m = 28$ probe-measurements: (Top Left) $n = 0$ (i.e. all $|+\rangle$ detection) as a function of the scaled measurement interval $\bar{g}\tau$. For larger values of $\bar{g}\tau$ we get a highly purified bath state, indicating the Anti-Zeno regime. (Top Right) Spectrum of a 4-spin-1/2 bath, for $m = 28$ measurements with $\bar{g}\tau = 0.8$ as a function of n – the number of $|-\rangle$ detections (Anti-Zeno regime). (Bottom) Spectrum of a 4-spin-1/2 bath, for $m = 28$ measurements with $\bar{g}\tau = 0.05$ as a function of n – the number of $|-\rangle$ detections (Zeno regime).

$$\rho_B^{\{n,(m-n)\}}(\tau) = \frac{1}{\mathcal{N}_{n,(m-n)}(\tau)} \sum_j P_j(0) \, \frac{1}{2^m} \left[1 + \cos\left(\frac{2\omega_j}{\bar{g}} \bar{g}\tau\right)\right]^{(m-n)} \left[1 - \cos\left(\frac{2\omega_j}{\bar{g}} \bar{g}\tau\right)\right]^n |j\rangle\langle j|, \tag{14}$$

we note that for $n = 0$, the only surviving factors on the r.h.s. of Supplementary Eq. (14) are of the form

$$\left[1 + \cos\left(\frac{2\omega_j}{\bar{g}} \bar{g}\tau\right)\right]^m. \tag{15}$$

In order to understand the nature of this spectrum for different values of $\bar{g}\tau$ we compare the behaviour of $\cos(x)$ and x in the range $0 \leq x \leq \pi$ as shown in Supplementary Fig.3. The graphical solution of $\cos(x) = x$ yields $x \approx 0.74$ for $x \geq 0$. For $|x| \ll 0.74$, $\cos(x) \to 1$. In Supplementary Fig.1, for $\bar{g}\tau = 0.8$, we have $2\left|\frac{\omega_j}{\bar{g}}\right|_{\min} \bar{g}\tau \sim 0.32 < 0.74$. Thus we observe high amplitudes of the post-measurement spectrum for this value. For all other values of

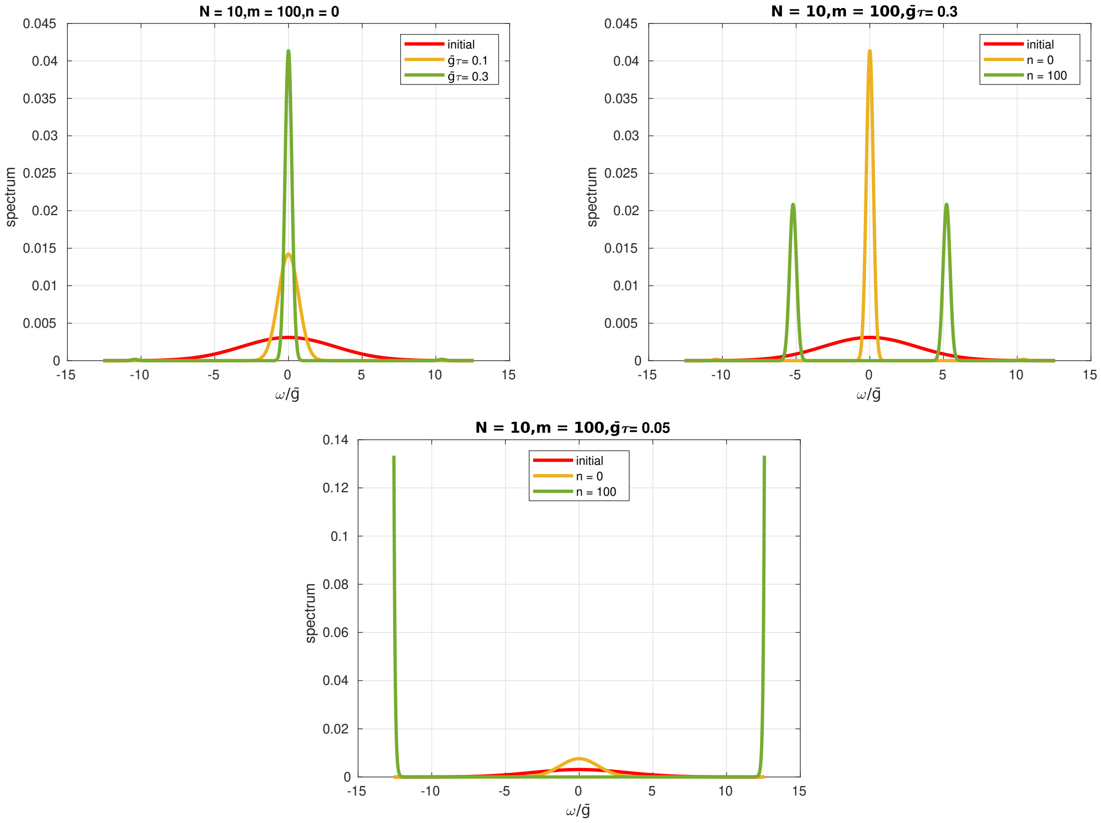

Figure Supplementary Fig.2. Spectrum, $P(\omega)$ of a 10-spin-1/2 bath, for $m = 100$ probe-measurements: (Top Left): (Top Left) $n = 0$ (i.e. all $|+\rangle$ detection) as a function of the scaled measurement interval $\bar{g}\tau$. For larger values of $\bar{g}\tau$ we get a highly purified bath state, indicating the Anti-Zeno regime. (Top Right) Spectrum of a 4-spin-1/2 bath, for $m = 100$ measurements with $\bar{g}\tau = 0.3$ as a function of n – the number of $|-\rangle$ detections (Anti-Zeno regime). (Bottom) Spectrum of a 4-spin-1/2 bath, for $m = 100$ measurements with $\bar{g}\tau = 0.05$ as a function of n – the number of $|-\rangle$ detection (Zeno regime).

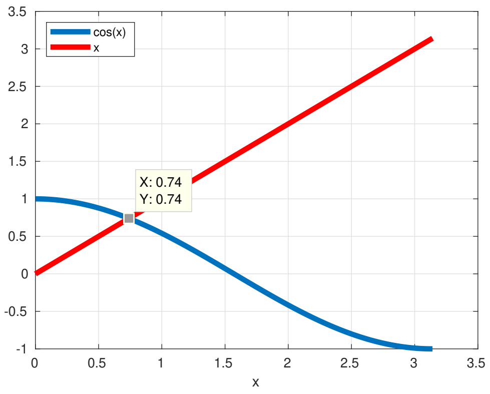

Figure Supplementary Fig.3. Intersection of $y = \cos(x)$ and $y = x$ in $0 \leq x \leq \pi$

$\left|\frac{\omega_j}{\bar{g}}\right|$ this condition is not satisfied leading to blackuced spectral-amplitudes. The overall

Gaussian envelope enhances the contrast between the peak and other values. This is the Anti-Zeno (AZE) regime of measurement intervals $\bar{g}\tau$.

The opposite Zeno (QZE) limit is obtained for $\bar{g}\tau \ll 0.74$. In this case for $n = 0$ (i.e. all $|+\rangle$ detection), in the QZE limit $\bar{g}\tau \to 0$, we have from Supplementary Eq. (14) and Supplementary Eq. (15)

$$\rho_B^{\{0,m\}}(\tau) \approx \sum_j P_j(0) \;\; |j\rangle\langle j| = \rho_B(0) \tag{16}$$

i.e. the bath spectrum remains unchanged (frozen) throughout the CT as shown in Supplementary Fig.1. This is the Zeno (QZE) regime of measurement intervals $\bar{g}\tau$.

Interestingly, in both the AZE and QZE regimes of the measurement intervals $\bar{g}\tau$ described above, the bath-spectra depend on the CT through n, the number of $|-\rangle$ detections. For $m = n$, the only surviving factors on the r.h.s. of Supplementary Eq. (14) are of the form

$$\left[1 - \cos\left(\frac{2\omega_j}{\bar{g}}\bar{g}\tau\right)\right]^n. \tag{17}$$

In this case we have negligible spectral contributions for $2\left|\frac{\omega_j}{\bar{g}}\right|\bar{g}\tau \to 0$. In Supplementary Fig.1, for $\bar{g}\tau = 0.05$ (QZE regime of $\bar{g}\tau$), the edge states have the highest non-negligible value: $2\left|\frac{\omega_j}{\bar{g}}\right|_{\max}\bar{g}\tau \sim 0.32 < 0.74$. Hence these states should have the highest spectral amplitudes. All other frequencies $\left|\frac{\omega_j}{\bar{g}}\right|$ have even lower values of the argument $2\left|\frac{\omega_j}{\bar{g}}\right|\bar{g}\tau$, leading to blackuced spectral amplitudes. Yet this does not fully explain the dramatic enhancement of the edge-state amplitudes observed in Supplementary Fig.1 and Supplementary Fig.2 in the QZE regime of $\bar{g}\tau$. Moreover, the overall Gaussian envelope indicates further blackuction of the edge-state amplitudes.

The key feature that results in the spectacular enhancement of the edge-state amplitudes in the QZE regime of $\bar{g}\tau$ is the normalization factor $\mathcal{N}_{n,0}(\tau)$, resulting from selective single-shot measurements. Since the major contribution to $\mathcal{N}_{n,0}(\tau)$ comes from the edge-states in this case, normalization with $\mathcal{N}_{n,0}(\tau)$ shoots up the edge-state spectral amplitudes resulting in the observed dramatic enhancement in Supplementary Fig.1 and Supplementary Fig.2.

For $\bar{g}\tau = 0.8$ and $m = n$ (AZE regime of $\bar{g}\tau$) in Supplementary Fig.1, $2\left|\frac{\omega_j}{\bar{g}}\right|_{\max}\bar{g}\tau \approx 5 \gg 0.74$. Thus in this case the edge-states may or may not have the highest contribution to the spectrum. Consequently, the spectral peaks appear at intermediate positions, jointly determined by the Gaussian envelope $P_j(0)$, the normalization factor $\mathcal{N}_{n,0}$ and the filter function $\left[1 - \cos\left(\frac{2\omega_j}{\bar{g}}\bar{g}\tau\right)\right]^n$.

Supplementary Note 4. FREE INDUCTION DECAY MODIFICATION IN THE ZENO AND ANTI-ZENO REGIMES

Here we calculate the Free Induction Decay (FID) signal obtained from probe measurements for a probe initialized to $|+\rangle$. Using the time evolution operator U_{S+B}, we can find the joint state of the probe and bath at any time t. Tracing out the bath variables, we obtain the probe density matrix $\rho_S(t)$. Projection of $\rho_S(t)$ on $|+\rangle\langle+|$ then results in the FID that expresses the coherence decay probability. Prior to the CT measurement sequence, this

probability is determined by the initial j-state probabilities $P_j(0)$ (see Supplementary Eq. (16)) as

$$p(t) = \sum_j P_j(0)\cos^2(\omega_j t). \tag{18}$$

Since the $P_j(0)$ are drawn from a Gaussian distribution, the FID is then the Gaussian average of $\cos^2\Big(\omega_j t\Big)$. Thus, the pre-measurement (prior) FID is given by

$$\begin{aligned} p_{\rm pre}(t) &= \Big\langle \cos^2\Big(\frac{\omega_j}{g} gt\Big)\Big\rangle_{G_0} \\ &= \frac{1}{2}\Big[1 + \exp\Big(-\frac{t}{T_2}\Big)^2\Big] \end{aligned} \tag{19}$$

where T_2 is a constant depending on ω_k-s, and other spectral parameters and $\Big\langle ...\Big\rangle_{G_0}$ denotes Gaussian averaging with zero mean.

Supplementary Eq. (19) holds exactly only for infinite ensembles. Since we are concerned with spin-bath ensembles of finite size, we shall use Supplementary Eq. (18) instead of Supplementary Eq. (19) to compute the pre-measurement (prior) FID. The pre-measurement FID resembles the $\exp\Big(-\frac{t}{T_2}\Big)^2$ decay law, with finite-size effects appearing at long times, which we ignore. Similarly, post-measurement (posterior) FID is obtained from Supplementary Eq. (18) by replacing $P_j(0)$ with the appropriate post-measurement distribution.

Supplementary Fig.4 shows the FID obtained in the Anti-Zeno regime for $n = 0$ (i.e. all $|+\rangle$ detection) and $n = 5$ (i.e. 5 $|-\rangle$ detections) in a sequence of $m = 28$, single-shot measurements. The black curve in Supplementary Fig.4 matches the experimentally obtained FID, shown in 2(b) of the manuscript. The green curve on the left panel of Supplementary Fig.4 has a damped oscillatory behaviour, which is not of the form $\exp\Big(-\frac{t}{T_2}\Big)^2\cos(\omega t)$. This becomes more evident if we calculate the corresponding curve for a larger value of $\bar{g}\tau$ as shown in the right panel of Supplementary Fig.4. The FID obtained in the Zeno-regime is also shown in Supplementary Fig.4. The FID obtained for an $N = 10$ spin-1/2 bath is shown in Supplementary Fig.5.

Supplementary Note 5. CORRELATION MEASUREMENTS

The probability of a $|+\rangle$-state detection at time t_d, long after the bath has reached a steady-state following the CT $M_{n,(m-n)}$, is

$$\begin{aligned} p_+(m\tau + t_d) &= {\rm Tr}\Big[|+\rangle\langle+|\,\rho(m\tau + t_d)\Big] \\ &= \sum_j \Big[\frac{1}{2^N} + e^{-\Gamma t_d}\Big\{P_j(m,n) - \frac{1}{2^N}\Big\}\Big]\cos^2(\omega_j t_d), \end{aligned} \tag{20}$$

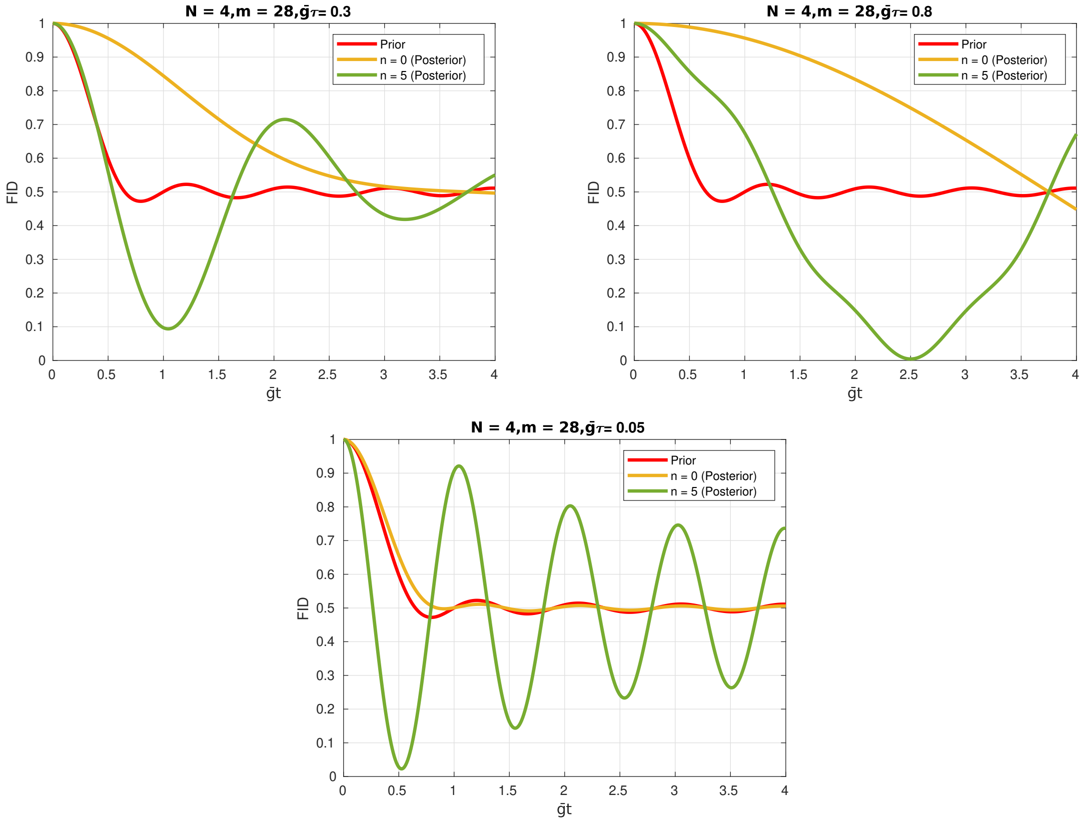

Figure Supplementary Fig.4. Probe FID for a 4-spin-1/2 bath with $m = 28$ measurements with $\bar{g}\tau = 0.3$ (top left), $\bar{g}\tau = 0.8$ (top right) and $\bar{g}\tau = 0.05$ (bottom), as a function of n – the number of $|-\rangle$ detection. $\bar{g}\tau = 0.8$ corresponds to the AZE regime. For $\bar{g}\tau = 0.05$ the QZE regime holds for the orange and black curves, and the AZE regime for the green curve.

where $P_j(m,n) = P_j(0)\ \cos^{2(m-n)}(\omega_j\tau)\ \sin^{2n}(\omega_j\tau)$. Similarly the probability of $|-\rangle$ detection is

$$p_-(m\tau + t_d) = \sum_j \left[\frac{1}{2^N} + e^{-\Gamma t_d}\left\{P_j(m,n) - \frac{1}{2^N}\right\}\right]\ \sin^2(\omega_j t_d). \tag{21}$$

At sufficiently long times, $t_d \geq \Gamma^{-1}$, the second terms in the expressions for $p_\pm$ become negligible. Thus, in this limit we have

$$p_\pm(m\tau + t_d) \approx \frac{1}{2^{N+1}}\ \sum_j \Big[1 \pm \cos(2\omega_j t_d)\Big]. \tag{22}$$

Supplementary Note 6. STEADY STATES OF THE BATH ENGINEEBLACK FOR QUANTUM SENSING

We have seen thus far (SI Sec. III-IV) that the Anti-Zeno (AZE) polarization of the bath in the absence of an external field and for a non-interacting spin bath leads to a maximum bath polarization of 0.5, on account of the $|\pm$ state symmetry of the probe. An example

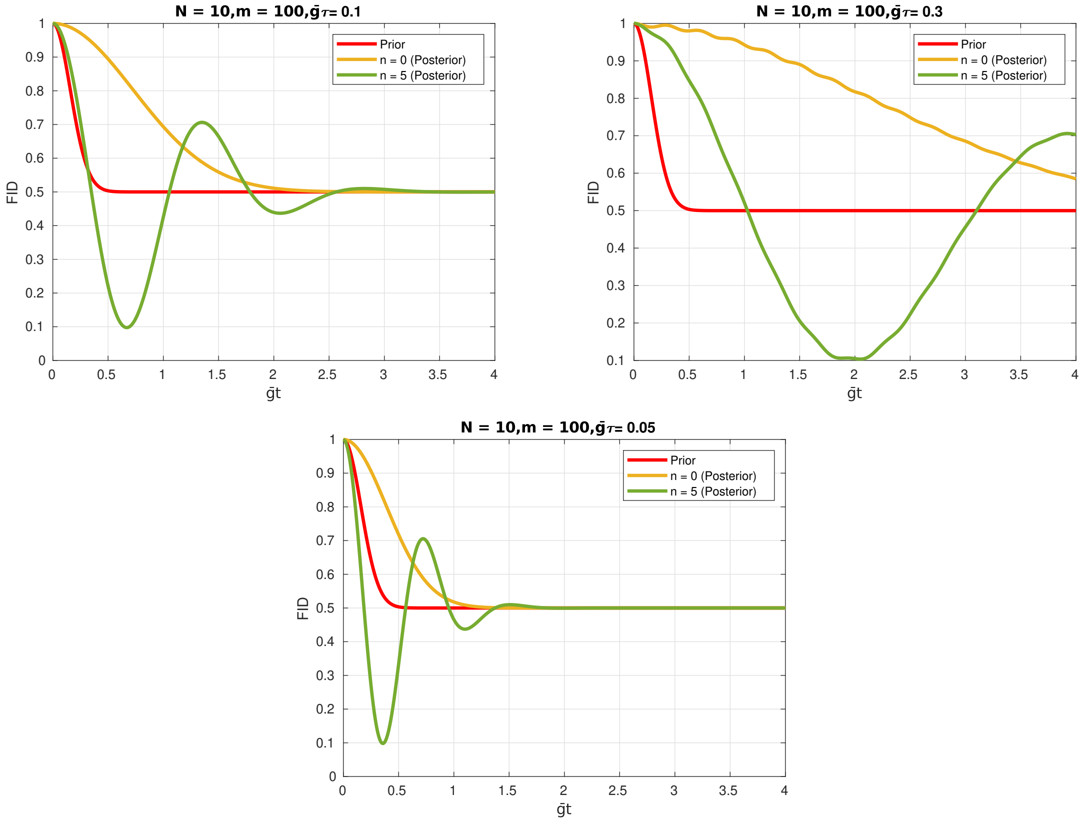

Figure Supplementary Fig.5. Probe FID for a 10-spin-1/2 bath with $m = 100$ probe-measurements with $\bar{g}\tau = 0.3$ (top left), $\bar{g}\tau = 0.3$ (top right) and $\bar{g}\tau = 0.05$ (bottom), as a function of n – the number of $|-\rangle$ detection.

is the polarized bath state shown in Fig. S3, where the polarized bath state is a statistical mixture in the highest angular momentum ($L = N/2$) subspace of the form

$$\rho_B = \frac{1}{2}[| + N/2\rangle\langle +N/2| + | - N/2\rangle\langle -N/2|]. \tag{23}$$

Such states have no practical application in quantum sensing. For sensing applications we may resort to the intra-bath couplings (SI Sec. I C, I D) that modify the probe-measurement CT's to achieve a much broader variety of steady states of the bath.

Remarkably, for the CT $\mathbf{M_{m,0}}$ acting on a spin-chain bath with $N = 4$ and uniform nearest-neighbor coupling J, we get a completely pure steady state of the bath that is useful for quantum sensing:

$$|\psi\rangle_B = \frac{1}{\sqrt{2^{N/2}}} \bigotimes_k [|01\rangle - |10\rangle]_{k,k+1}. \tag{24}$$

In contrast, for the CT $\mathbf{M_{0,n}}$ we obtain the mixed state as in Eq. (S21).

The purity of this bath for the advantageous CT $\mathbf{M_{m,0}}$ with $g_1\tau = \pi$ is shown in Supplementary Fig.6. The corresponding bath density matrix is shown in Supplementary Fig.7.

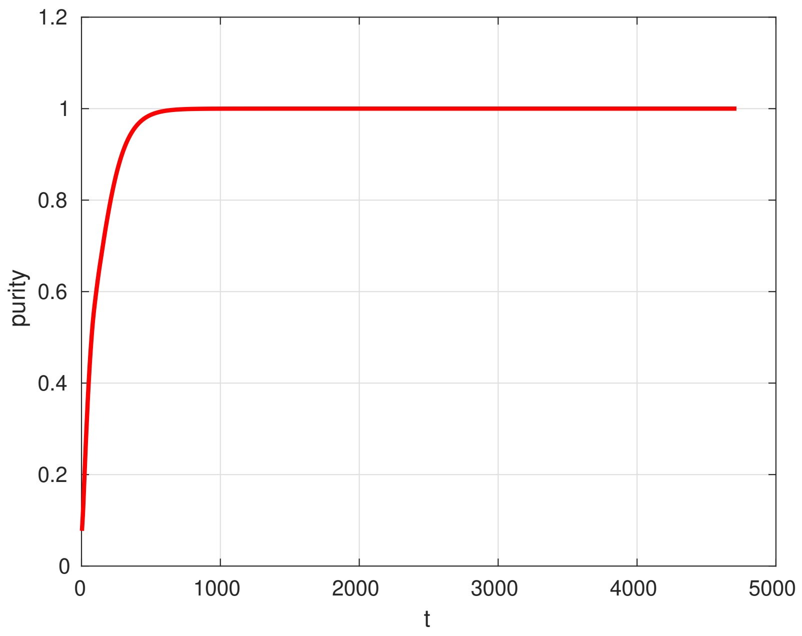

Figure Supplementary Fig.6. Purity of open chain bath with $N = 4$ and uniform nearest neighbour coupling $J = 2$ as a function of time for CT $\mathbf{M_{m,0}}$ and $\bar{g}\tau = \pi$.

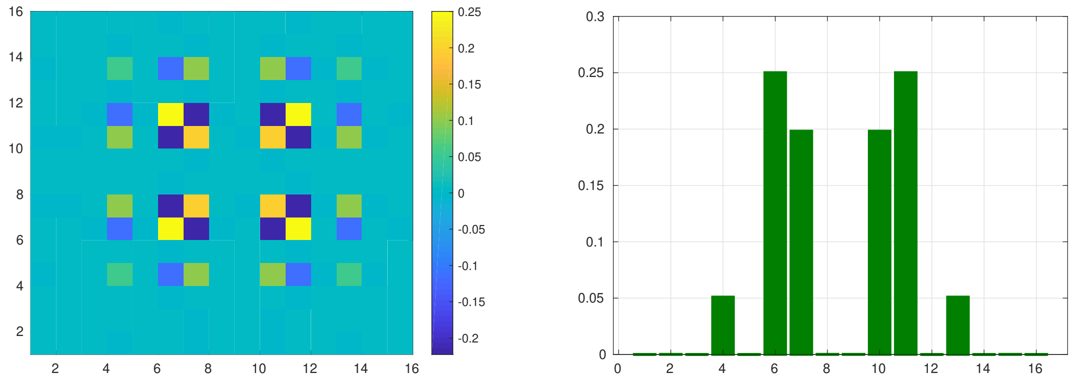

Figure Supplementary Fig.7. Steady-state density matrix of open chain bath with $N = 4$ and uniform nearest neighbour coupling $J = 2$ corresponding to CT $\mathbf{M_{m,0}}$ and $\bar{g}\tau = \pi$. The left panel shows the full density matrix profile, while the right panel shows the relative magnitudes of the diagonal elements of the steady-state density matrix.

To obtain full purity of the bath, one can switch on resonant polarization transfer between the probe and bath spins according to the Hartmann-Hahn procedure [3–5] and then perform a selective measurement on the probe that can polarize the bath completely. If the bath is in the $|N/2\rangle$ state, which is an eigenstate of the resonant S-B (flip-flop) interaction, the probe state remains undisturbed and hence a selective measurement will lead to a deterministic measurement result collapsing the bath to the fully polarized $|N/2\rangle = |0, 0 \cdots 000\rangle$ state.

This fully polarized state, as well as the state given in Eq. (S23), can be readily used in quantum sensing protocols.

SUPPLEMENTARY REFERENCES

---

[1] London, P. and Scheuer, J. and Cai, J.-M. and Schwarz, I. and Retzker, A. and Plenio, M. B. and Katagiri, M. and Teraji, T. and Koizumi, S. and Isoya, J. and Fischer, R. and McGuinness, L. P. and Naydenov, B. and Jelezko, F. *Phys. Rev. Lett.* **111**, 067601, (2013). [Supplementary Information].

[2] Zwick, A., Álvarez, G. A., Bensky, G. & Kurizki, G. Optimized dynamical control of state transfer through noisy spin chains. *New J. Phys.* **16**, 065021, (2014).

[3] Hartmann, S. R. and Hahn, E. L. Nuclear Double Resonance in the Rotating Frame. *Phys. Rev.* **128**, 2042–2053, (1962).

[4] Slichter, C. P. *Principles of Magnetic Resonance* (Springer Science & Business Media, New York, 2013).

[5] Rao, D.D.B., Ghosh, A., Gelbwaser-Klimovsky, D., Bar-Gill, N. & Kurizki, G. Spin-bath polarization via disentanglement. *New J. Phys.* **22**, 083035, (2020).
